# Hybrid Intracavitary-Interstitial brachytherapy in a case of nasal vestibule cancer penetrating the hard palate

**DOI:** 10.1259/bjrcr.20200178

**Published:** 2021-03-25

**Authors:** Naoya Murakami, Go Omura, Wakako Yatsuoka, Hiroyuki Okamoto, Seiichi Yoshimoto, Takao Ueno, Jun Itami

**Affiliations:** 1 Department of Radiation Oncology, National Cancer Center Hospital, 5-1-1 Tsukiji, Chuo-ku, Tokyo, Japan; 2 Department of Head and Neck Surgery, National Cancer Center Hospital, 5-1-1 Tsukiji, Chuo-ku, Tokyo, Japan; 3 Dental Division, National Cancer Center Hospital, 5-1-1 Tsukiji, Chuo-ku, Tokyo, Japan; 4 Department of Medical Physics, National Cancer Center Hospital, Tsukiji 5-1-1, Chuo-ku, Tokyo, Japan

## Abstract

Because of its rarity, no standard therapy exists for localized squamous cell carcinoma of the nasal vestibule. Interstitial brachytherapy (ISBT) is reported to be a preferable treatment modality of choice for early-stage localized nasal vestibule cancer. In this report, a nasal vestibule cancer with hard palate invasion (T3) was treated by definitive radiation therapy. Because it was considered to be difficult to cover the entire target volume only by ISBT, a hybrid of intracavitary (dental mold-based) and ISBT was applied to the patient following external beam radiation therapy.

Nasal vestibule cancer is a rare head and neck cancer entity. According to the Monitoring of Cancer Incidence in Japan, a nation-wide cancer statistic in Japan, the incidence of the nasal cavity and middle ear cancer in 2015 was 702 in Japan.^1^ Because of its rarity, the incidence of nasal cancer is counted together with the middle ear in Japan, therefore, there is no statistical data only for nasal cancer incidence in Japan. Again, because of its rarity, there is no standard therapy for localized nasal vestibule cancer. Surgery and external beam radiation therapy (EBRT) are mostly performed for localized nasal vestibule cancer. For nasal vestibule cancer, Wang’s classification^2^ is reported to predict prognosis better than the other staging classification systems.^3^ For Wang T1-2 disease, the prognosis of patients treated by EBRT was favorable between 3-y progression-free survival of 71–83%, while that of Wang T3 was reported to be 50%.^2^ Therefore, for Wang T3 disease, radical surgery followed by reconstruction plastic surgery with or without post-operative EBRT can be offered, however, it is at the cost of poor cosmetic appearance. Interstitial brachytherapy (ISBT) has been performed for nasal vestibule cancer with comparable oncologic results with surgery and better aesthetic outcomes.^4–7^ In this technical note, the authors successfully treated a Wang T3N0 nasal vestibule patient having bone involvement in the hard palate with a hybrid of intracavitary (dental mold-based) and ISBT (defined as hybrid brachytherapy, HBT). Written informed consent was obtained from the patient and this report was approved by the Institutional Review Board of National Cancer Center Hospital (the approved number is 2017-091) according to the ethical standards laid down in the Declaration of Helsinki.

## Purpose

### Case and technical description

**Figure 1.**
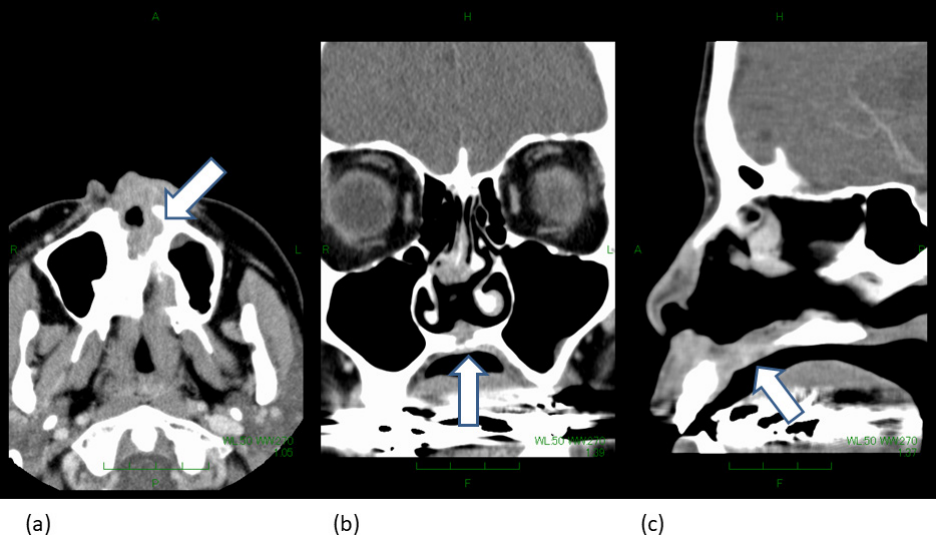
The figure shows pretreatment computed tomography of the patient with nasal vestibule cancer. (**a**), (**b**), and (**c**) represents the axial, coronal, and sagittal image, respectively. The white arrow represents a part where the tumor involves the bony structure of the hard palate.

**Figure 2.**
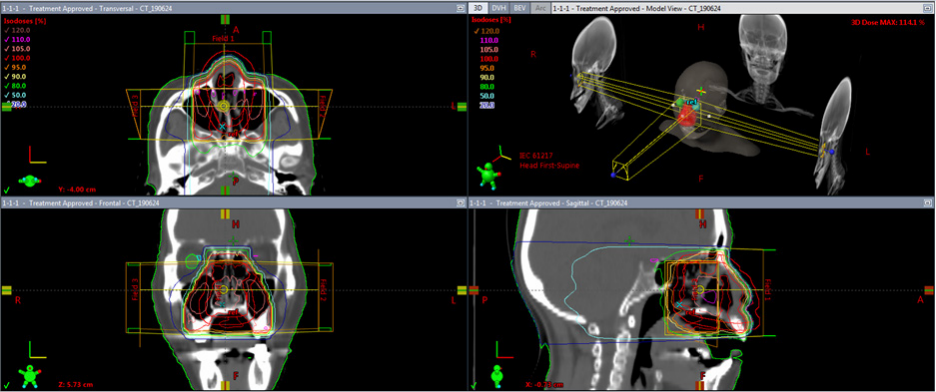
The figure shows the dose distribution of three-dimensional conformal radiation therapy with three portals. The entire nasal cavity was covered by the red isodose line represents 100% of the prescribed dose. Note that the patient’s mouth was kept open with a dental mouthpiece shown in Figure 3.

**Figure 3.**
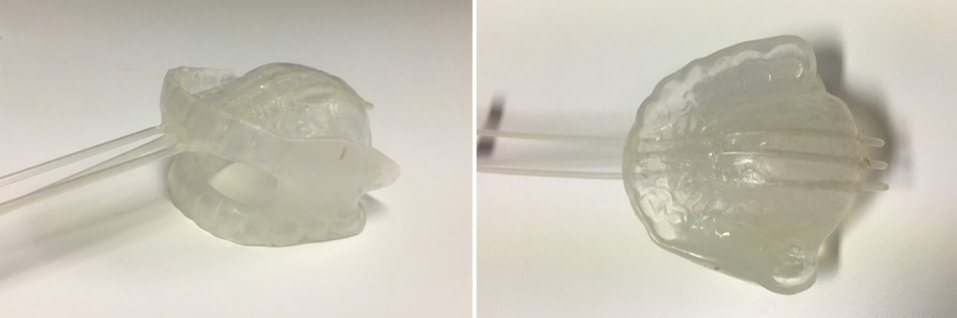
A mouth piece was crafted by dental plastic to keep the mouth open during radiation therapy. After completion of the external beam radiation therapy, three catheters were mounted on the mouth piece to improve the dose coverage of the tumor especially a part involving the hard palate through intracavitary brachytherapy together with the interstitial irradiation through the interstitial needles.

**Figure 4.**
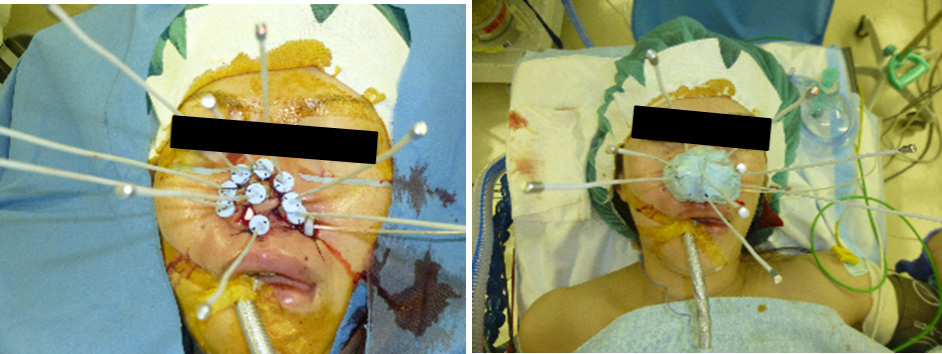
The figure shows macroscopic images of the interstitial brachytherapy implant. Under general anesthesia a total of ten 5 French ProGuide® sharp plastic needles (Nucletron, an Elekta company, Elekta AB, Stockholm, Sweden) were inserted. Each needle was fixed with a button sewed to the surface of the nose.

**Figure 5.**
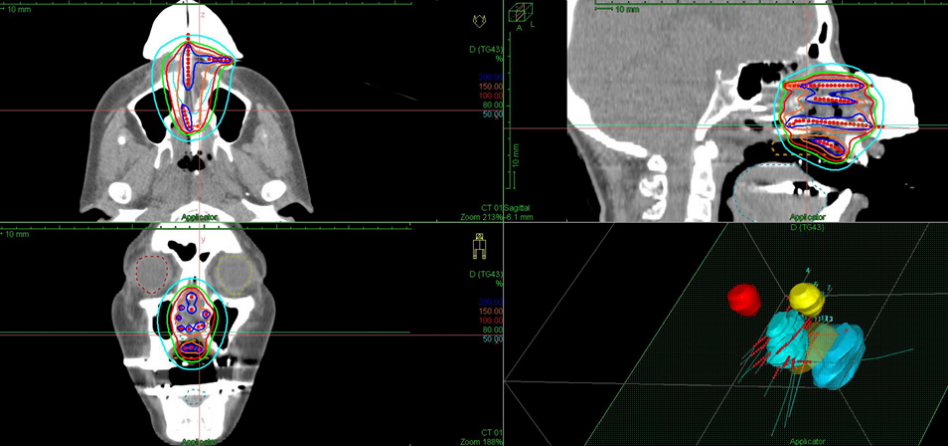
The figure shows the isodose distribution of the hybrid intracavitary-interstitial brachytherapy (HBT) implant. Blue, orange, red and light-green lines represent the 200%-, 150%-, 100%-, and 80%-isodose lines, respectively. Note that the invaded part of the hard palate was adequately covered by the 100% isodose line.

**Figure 6.**
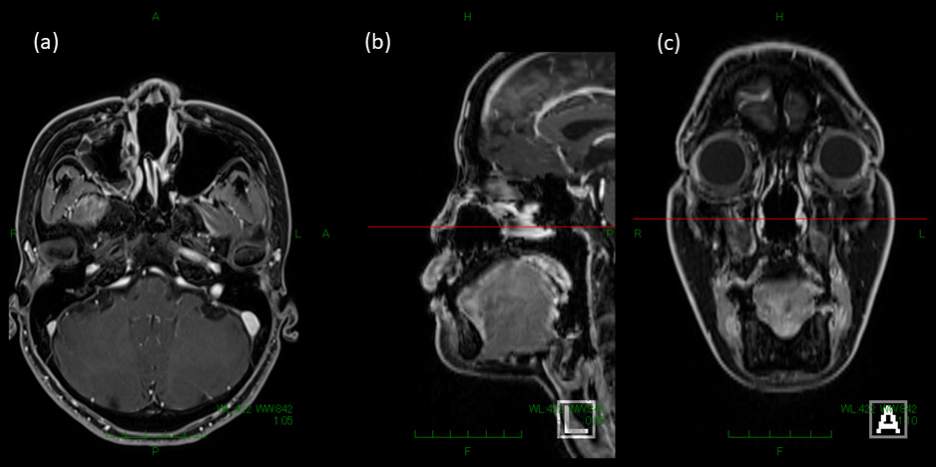
The figure shows magnetic resonance images taken 15 months after the completion of radiation therapy. (**a**), (**b**), and (**c**) represents the axial, sagittal, and coronal image, respectively. No obvious residual tumor was noted.

**Figure 7.**
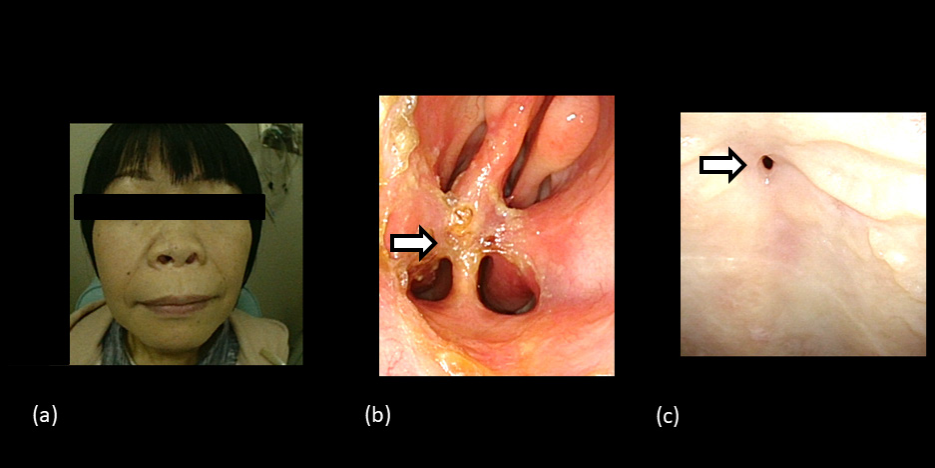
Images taken by flexible fiberscope 15 months after the completion of radiation therapy. (**a**) shows slight lowering of the nose. (**b**) shows adhesion of the posterior part of the nasal cavity requiring frequent dilatation. (**c**) shows a fistula in the hard palate.

A 51-year-old otherwise healthy female suffered from Wang T3N0M0, Stage III squamous cell carcinoma of the nasal vestibule.^2^ The reason for being T3 was the direct bone invasion to the hard palate. At the initial presentation, the frontal part of the nasal septum was largely eroded by the tumor and the bilateral nasal cavities became a common cavity (Figure 1). To preserve the esthetic appearance, she chose to receive primary radiotherapy. Because clinically no lymph node metastasis was noted before treatment, no treatment was performed to the regional neck lymph node basin. A combination of EBRT and brachytherapy as a boost was offered. Initially, 40 Gy in 20 fractions of EBRT by the three-dimensional conformal radiation therapy with three portals was given (Figure 2). To prevent the floor of the mouth from unnecessary irradiation exposure, our dental team created a customized mouthpiece to keep the mouth open during EBRT (Figure 3). Both dermatitis and mucositis after EBRT assessed by the Common Terminology Criteria for Adverse Events version 5.0 were Grade 1. The next Monday following the last session of EBRT, interstitial needle insertion was performed under general anesthesia (Figure 4). The depth of the needles was guided by X-ray fluoroscopy. A total of ten 5 French ProGuide® sharp plastic flexible needles (Nucletron, an Elekta company, Elekta AB, Stockholm, Sweden) were inserted and each needle was fixed with a button sewed to the surface of the nose. Because it was difficult to insert needles through the mouth to cover the hard palate invasion, three plastic catheters were mounted on the mouthpiece which was used during EBRT and used as a mold (Figure 3). Using interstitial needles and catheters mounted on the mouthpiece, HBT was performed. Treatment planning was based on CT images of 1 mm slice interval (Aquilion^™^ LB CT scanner, Canon Medical System, Japan). Dose calculation was performed using Oncentra Brachy v. 4.5.1 (Nucletron, an Elekta company, Elekta AB, Stockholm, Sweden) so that the 100% isodose line covered the clinical target volume (CTV) defined on the CT referring pre-treatment MRI and PET and CTV-D90 (dose covering at least 90% of the CTV) became larger than the prescribed dose based on CT image (image-guided brachytherapy) (Figure 5). The prescribed dose per fraction was 4 Gy and a total physical dose of 28 Gy in 7 fractions in 4 days was delivered. The needle insertion was performed once and irradiation was conducted bid at a 6 h interval. Interstitial needle insertion and removal were performed without any severe complications. At the first visit a week after the completion of the HBT, fistula formation in the hard palate was noted. The worst dermatitis and mucositis were Grade 1 and 2, respectively. Three months after the treatment, the patient experienced synechiae of the nasal cavity which can easily be managed by frequent dilatation of the narrowed cavity. Magnetic resonance images and fiberscope pictures taken 15 months after the completion of HBT showed no residual tumor with no late severe toxicity except a pinhole perforation in the hard palate and slight lowering of the nose (Figures 6 and 7). This patient was content with the clinical outcomes of this treatment at least at the time point when this article is written, except for the hardest 4 days when she was hospitalized with needles in place. Grade 2 nasal pain was noted during needles in place, but no continuous opioid administration was required. The worst acute mucositis after completion of whole radiation therapy was grade 2, requiring non-steroidal anti-inflammatory drugs.

## Discussion

Along with surgery, EBRT alone is frequently selected for treating nasal vestibule cancer. For Wang T1-2 disease, the prognosis of patients treated by EBRT is favorable between 3-y progression-free survival of 71–83%, however, that of Wang T3 disease is unsatisfactory low as 50%.^2^ There are several reports regarding favorable oncologic and cosmetic outcomes for patients with T1-2 nasal vestibule cancer treated with ISBT,^4–7^ but for T3 disease, usually surgery followed by EBRT is recommended.^2^ Do et al reported that five-year locoregional control for locally advanced T4 head and neck cancer with bone and cartilage invasion treated by definitive chemoradiotherapy was as low as 43%.^8^ Samant et al investigated clinical outcomes for 135 T4 head and neck cancer patients with or without bone or cartilage invasion treated by concomitant intra-arterial cisplatin and radiation therapy.^9^ Although no statistically significant difference was noted, they found a trend that lower response rates in cases with bone invasion (58.6%) compared to those with cartilage invasion (81.2%). Wang et al reported the clinical outcome of 36 nasal vestibular cancer patients, including six T3 patients treated by radiation therapy with or without ISBT.^2^ Complications were infrequent, and one patient developed fistula formation between the hard palate and soft palate; however, three-year progression-free survival was as low as 50%. Therefore, head and neck cancer patients with bone or cartilage invasion have been generally treated by primary surgery followed by adjuvant radiotherapy. In this technical report, the authors successfully treated a Wang T3N0 nasal vestibule patient having bone involvement in the hard palate with HBT. Together with our dental team, we previously reported a technique of HBT for treating localized buccal mucosa cancer,^10^ therefore, it was considered that a similar technique could be applied for the current patient to cover the hard palate invasion which was difficult to cover only by interstitial needles. Although the patient experienced fistula in the hard palate, the direct hard palate bone invasion was already present at the initial presentation, therefore, it is unlikely that this fistula formation was attributed solely by HBT. Because no prophylactic treatment was performed to the clinically negative neck lymph node area, further follow up is needed. Currently, no inhomogeneity is considered in the dose calculation of brachytherapy. Because the nasal cavity contains air space, a more sophisticated dose calculation algorithm is warranted to obtain more accurate dose calculation in the future.^11,12^

To the best of our knowledge, this is the first report of HBT for nasal vestibule cancer. In the management of head and neck cancer, ISBT has been used to escalate the local dose to obtain optimal local control,^13–20^ however, there are anatomic sites where it is difficult to insert and immobilize interstitial needles. In such a situation, HBT using mold can be a favorable modality of choice. In this technical note, a combination of intracavitary and ISBT was applied for T3 disease which penetrated the hard palate. Although primary surgery followed by EBRT remains standard of care for most T3 nasal vestibular cancers at the cost of poor cosmetic outcomes, the authors believe that with this novel HBT technique, widening of application of ISBT is possible for managing a group of T3 nasal vestibule cancer patients. The authors will continue to use the HBT technique for head and neck cancer in cases where it is difficult to cover the entire target volume only by interstitial needles.
